# Dampening HOTAIR sensitizes the gastric cancer cells to oxaliplatin through miR‐195‐5p and ABCG2 pathway

**DOI:** 10.1111/jcmm.17925

**Published:** 2023-08-24

**Authors:** Yaomin Luo, Xintong Lu, Wenrong Ma, Yang Xiao, Chen Wei, Xiaoxia Yuan, Yueyue Wu, Yunlin Wang, Yiman Xiong, Xin Yu, Xue Wu, Siqi He, Yayudie Liu, Jinjing Wang, Qing Wu, Hui Zhou, Zhen Jiang

**Affiliations:** ^1^ Institute of Basic Medicine and Forensic Medicine North Sichuan Medical College Nanchong China; ^2^ Department of Biochemistry and Molecular Biology, School of Basic Medicine and Forensic Medicine North Sichuan Medical College Nanchong China; ^3^ School of Pharmacy North Sichuan Medical College Nanchong China; ^4^ Department of Rehabilitation Medicine the Affiliated Hospital of North Sichuan Medical College Nanchong China; ^5^ Department of Clinical Laboratory the Affiliated Hospital of Jiaxing University Jiaxing China

**Keywords:** ABCG2, chemoresistance, gastric cancer, HOTAIR

## Abstract

Long non‐coding RNAs (lncRNA) have an extensive role in the progression and chemoresistance of gastric cancer (GC). Deeply study the regulatory role of lncRNAs could provide potential therapeutic targets. The aim of this study is to explore the regulatory role of HOTAIR in the progression and oxaliplatin resistance of GC. The expression of HOTAIR in GC and cell lines were detected by using qRT‐PCR. Cell proliferation and apoptosis were analysed by CCK‐8, EdU incorporation and flow cytometry. Luciferase reporter assay was used to identify the interaction between HOTAIR and ABCG2 (ATP‐binding cassette (ABC) superfamily G member 2, ABCG2) via miR‐195‐5p. The regulatory functions were verified by using molecular biology experiments. HOTAIR was significantly overexpressed in GC and associated with poor prognosis. Knock‐down of HOTAIR inhibited the GC cells proliferation and oxaliplatin resistance, while overexpression of HOTAIR showed opposite functions. Further studies found that HOTAIR acted as a competing endogenous RNA (ceRNA) to absorb miR‐195‐5p and elevated the expression of ABCG2, which leads to resistance of GC cells to oxaliplatin. Taken together, our findings demonstrated that HOTAIR regulates ABCG2 induced resistance of GC to oxaliplatin through miR‐195‐5p signalling and illustrate the great potential of developing new therapeutic targets for GC patients.

## 1 INTRODUCTION

Gastric cancer (GC) is one of the most common malignancies and the second leading cause of cancer‐related death worldwide.^1^ When at time of diagnosis, GC always develops to huge tumour burden, lymph node metastasis and vascular invasion due to its concealed onset. Currently, surgical resection in combination with chemotherapy remains the major treatment of GC.

Oxaliplatin (L‐OHP), a diaminocyclohexane (DACH) platinum, is the third generation platinum anticancer agency after cisplatin and carboplatin. Currently, oxaliplatin is the first‐line chemotherapy agency against GC by blocking DNA replication and transcription through crosslinking platinum atoms with DNA strands, thus producing anticancer activity.^2^, ^3^, ^4^ A number of studies confirmed the effectiveness of oxaliplatin in treatment of GC.^5^, ^6^, ^7^ However, as a monotherapy, only 20%–24% response rates as a first‐line therapy and 10% as a second‐line therapy was obtained in patients refractory to, or progressing after, fluorouracil‐based therapy in colon cancer.^2^ It was urgently to identify novel molecules that involved in these progresses.

Long non‐coding RNAs (lncRNA) are a class of non‐coding RNAs that longer than 200 nt and have limited or no capacity of protein coding. lncRNAs presented diverse function in regulating complex networks in the carcinogenesis of many types of cancers.^8^, ^9^, ^10^ LncRNA HOX transcript antisense RNA (HOTAIR) was first discovered to contribute the carcinogenesis of breast cancer through interacting with polycomb repressive complex 2 (PRC2).^11^ During the past years, a number of evidences have demonstrated that HOTAIR function as an important regulator in the development and metastasis of many types of cancer.^12^, ^13^ Although these studies revealed some aspects of HOTAIR function in cancer, the effect of HOTAIR on oxaliplatin resistance of GC remains poorly understood.

In this study, we demonstrated that HOTAIR was upregulated in GC and associated with oxaliplatin resistance. HOTAIR sponges miR‐195‐5p to elevate ABCG2 expression and promote oxaliplatin resistance. The present study demonstrated the regulatory mechanism of HOTAIR and provides potential therapeutic targets for GC patients.

## 2 MATERIALS AND METHODS

### 2.1 Cell culture and GC tissues

The human gastric cancer cell line AGS, HGC‐27 were cultured in RPMI‐1640 (Thermo Fisher Scientific) supplemented with 10% foetal bovine serum (Gibco) in an incubator sustaining an atmosphere of 37°Cand 5% CO_2_. The GC tissues and normal tissues were obtained from GC patients who received gastrectomy in North Sichuan Medical College (NSMC). The Ethics Committee of the NSMC approved all the aspects of this study.

### 2.2 Cell transfection

The Pex3 vector containing HOTAIR and the empty vector, miR‐195‐5p mimics and inhibitor, and shRNA against HOTAIR and sh‐NC were transfected into cells with Lipofectamine 2000 (GenePharma Company, Shanghai, China).

### 2.3 RNA extraction and qRT‐PCR

Isolation of total RNA was conducted by TRIzol reagent (Invitrogen). The quality and quantity of RNA samples were assessed by measuring the absorbance at 260 nm/280 nm (A260/280) using Nanodrop 2000. The reverse transcription was conducted by Reversed cDNA Synthesis Kit (Promega, Madison, WI, USA). qPCR was carried out on the Bio‐Rad referring to the manufactures' instructions. The gene expression was calculated based on the 2^−ΔΔCt^ method. The primer sequences were as follows: forward primer 5′‐ACTCTGACTCGCCTGTGCTCTG‐3′ and reverse primer 5′‐AGTGCCTGGTGCTCTCTTACCC‐3′ for HOTAIR.

### 2.4 Western blotting

Total protein was extracted with RIPA buffer supplemented with protease and phosphatase inhibitors. The concentration of protein was determined by using a BCA kit (Thermo Scientific, USA). The anti‐ABCG2 antibody used in this study was obtained from Beyotime Biotechnology (Hangzhou, China). Bands were normalised to β‐actin expression (Beyotime Biotechnology).

### 2.5 Cell viability assay

Cells were plated in 96‐wells plates at 1 × 10^3^ per well. After 24 h, cells were exposed to different concentrations of oxaliplatin and proceed to grow 96 h, then cells survival was determined by the Cell Counting Kit‐8 referring to the manufacture's guideline (Beyotime Biotechnology). The methods for analysis the cell viability were as follows: Inhibition rate (%) = (Negative control group − Experimental group)/Negative control group × 100%.

### 2.6 EdU incorporation assay

Cells were cultured in 24‐well plates for 24 h and then exposed to agents for 48 h. 10 μM EdU was added to each well and the cells were cultured for 2 h at incubator. Cells were fixed with 4% formaldehyde for 20 min at room temperature. Washed with PBS twice, the incorporated EdU was reacted with a Click‐iT EdU kit for 30 min at room temperature. Hoechst were added into wells for 20 min. Cells were visualised under a fluorescence microscope (Nikon, Minato City, Tokyo, Japan). The EdU incorporation rates = the EdU‐positive cells (red cells)/the Hoechst 33342‐positive cells (blue cells).

### 2.7 Apoptosis analysis

GC cells were seeded in 6‐well plates. Cells were added with plasmids and oxaliplatin for 48 h, then washed with PBS and harvested by trypsinization. Analysis of the cell apoptosis was performed as previously described.^14^ For apoptosis analysis, each group was tested in triplicate.

### 2.8 Luciferase assay

GC cells were seeded at 5 × 10^4^ cells/well in 24‐well plates. twenty‐four hour later, control miRNAs or miR‐195‐5p and 0.5 μg GP‐miRGLO luciferase reporter containing WT and Mut HOTAIR or ABCG2 3′‐UTR (GenePharma, Shanghai, China) were transfected into cells using lipofectamine 2000 (Thermo Fisher Scientific Inc., Waltham, MA, USA). After 48 h, luciferase activity was measured using a dual luciferase reporter assay kit (GenePharma) referring to the manufacturer's guideline. All tests were conducted in triplicate and repeated three times.

## 3 RESULTS

### 3.1 HOTAIR was upregulated in GC and associated with poor prognosis

**FIGURE 1:**
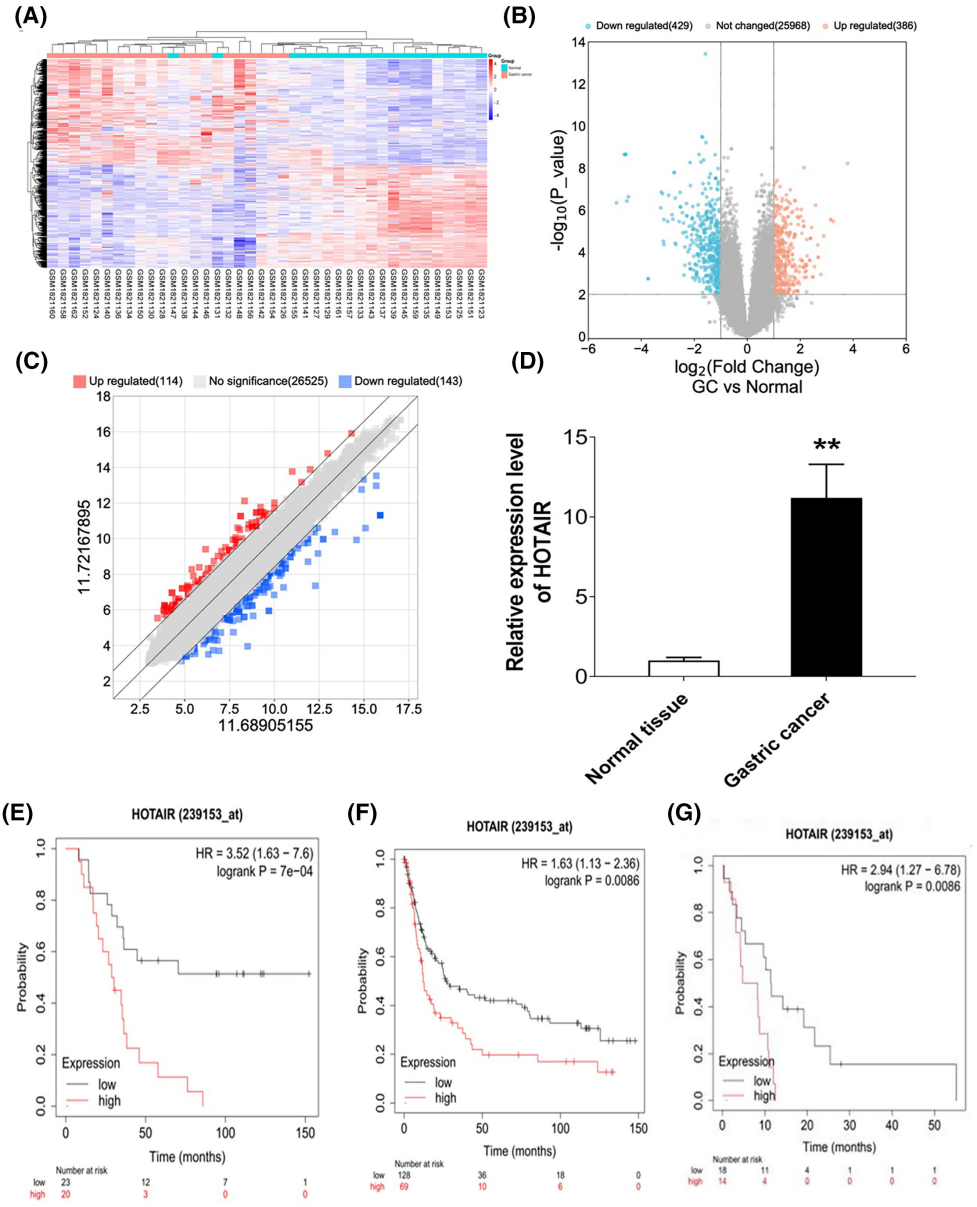
HOTAIR was highly expressed in GC and correlated with poor prognosis. (A–C) Heat map, volcano and scatter map of the significantly differentially expressed lncRNAs between 20 pairs of normal tissues and GC tissues (GSE70880, fold change≥2; *p* ≤ 0.05). HOTAIR was overexpressed with fold change of 2.06 in gastric cancer. (D) Relative expression of HOTAIR in GC and normal tissues (*n* = 50). (E–G) Prognosis analysis of overall survival (E), progression‐free survival (F) and post progression survival (G) in HOTAIR high expression and low expression group from Kaplan–Meier database.

To find the different expression of lncRNAs in GC, GSE70880 data were downloaded from the GEO database. 26,783 genes were included in this dataset. |Log FC| > 2.0 and FDR <0.05 was set to find the differentially expressed lncRNAs in GC compared with that in normal tissues. The data set contained 815 differentially expressed genes, including 386 upregulated genes and 429 downregulated genes. HOTAIR was found to be upregulated with logFC 2.06 in GC compared with that in normal gastric tissues (Figure 1A–C). Moreover, HOTAIR was found to be significantly upregulated in 50 GC patients compared with that in normal tissues (Figure 1D). We investigated the prognostic values of HOTAIR with GC patients using Kaplan–Meier database. The results showed that the patients with higher expression of HOTAIR presented worse overall survival (OS), progression‐free survival (PFS), and post progression survival (PPS) (Figure 1E–G).

### 3.2 HOTAIR promotes GC cell proliferation

**FIGURE 2:**
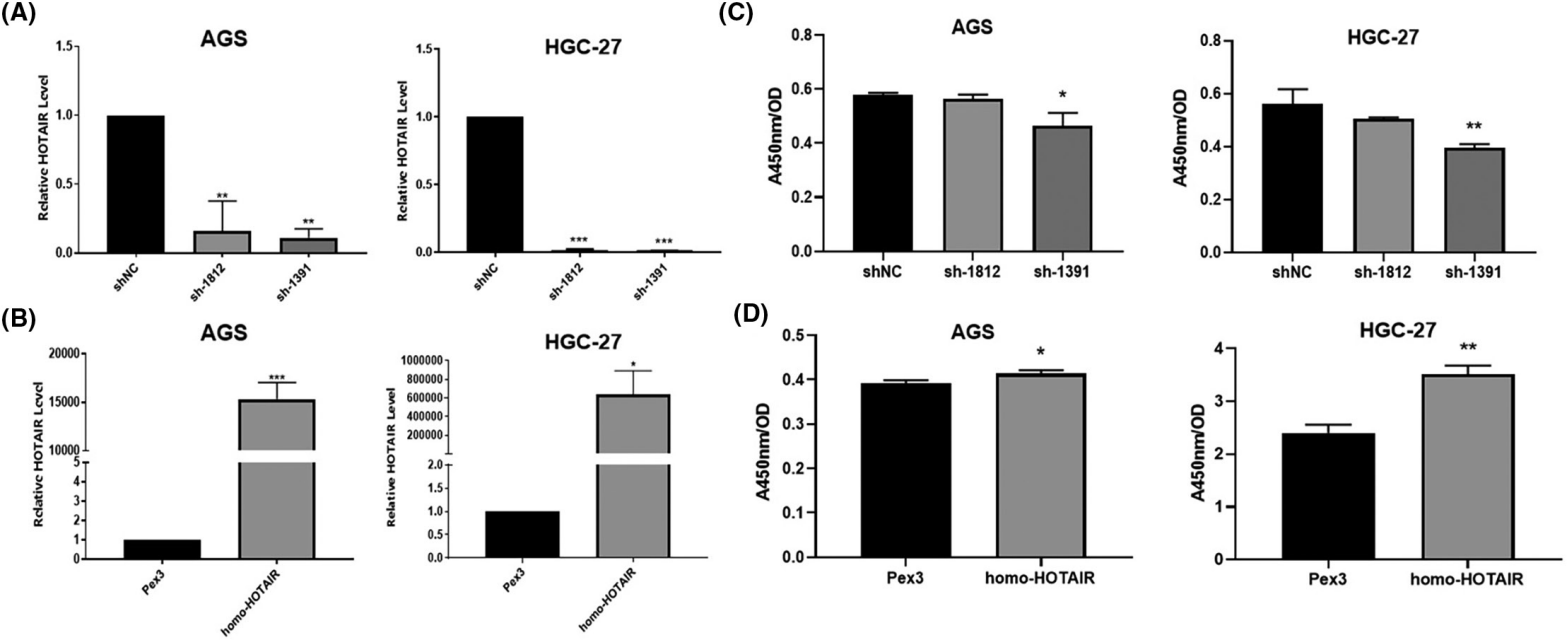
HOTAIR promotes gastric cancer cells proliferation. (A, B) The efficacy of knockdown and overexpression of HOTAIR. (C, D) CCK‐8 assay of AGS and HGC‐27 cells after knockdown (C) and overexpression (D) of HOTAIR. Data were expressed as the mean ± SD (*p < 0.05; ** *p <* 0.01; *** *p <* 0.001).

**FIGURE 3:**
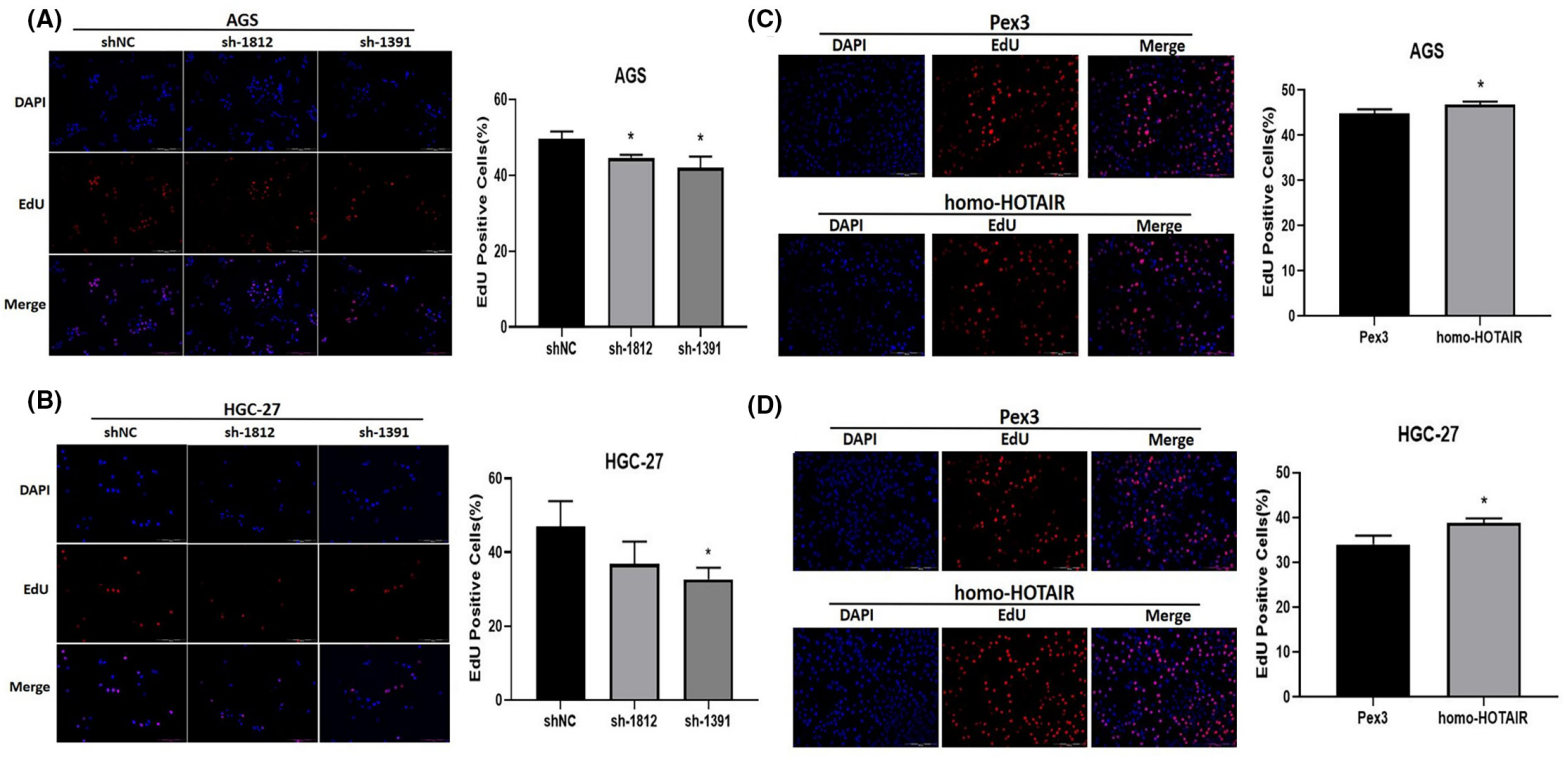
EdU assay of AGS and HGC‐27 cells with knockdown (A, B) and overexpression(C, D) of HOTAIR. Data were expressed as mean ± SD (*p < 0.05).

**FIGURE 4:**
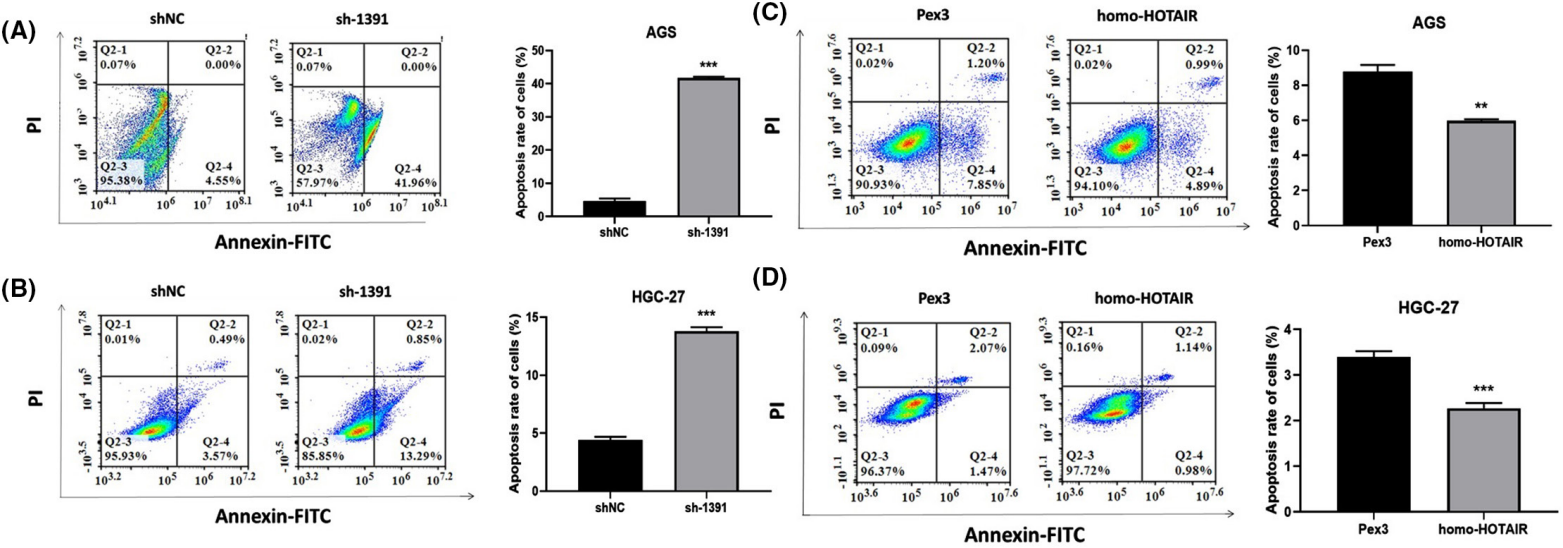
Apoptosis assay of AGS and HGC‐27 with HOTAIR knockdown (A, B) and overexpression (C, D). Data were expressed as mean ± SD (**p < 0.01; ***p < 0.001).

We tested several GC cell lines and found that HOTAIR was upregulated in AGS, HGC‐27. We transfected AGS, HGC‐27with shRNA against HOTAIR (sh‐1812 and sh‐1391) and HOTAIR overexpression plasmid (homo‐HOTAIR). The efficacy of transfection was tested by qRT‐PCR (Figure 2A,B). CCK‐8 assay showed that the proliferation of GC cells were significantly dampened after transfected with sh‐1812 and sh‐1391 compared to those were transfected with sh‐NC (Figure 2C), correspondingly the proliferation of GC cells were significantly accelerated after transfected with homo‐HOTAIR compared to those transfected with Pex3 (Figure 2D). In addition, silencing HOTAIR inhibited the EdU incorporation into GC cells while overexpression of HOTAIR enhanced those cell behaviours (Figure 3A–D). Moreover, the apoptosis rates of AGS and HGC‐27 were significantly elevated after HOTAIR knockdown but dampened after HOTAIR overexpression (Figure 4A–D, ** *p <* 0.01;*** *p <* 0.001).

### 3.3 HOTAIR promotes the resistance of GC cells to oxaliplatin

**FIGURE 5:**
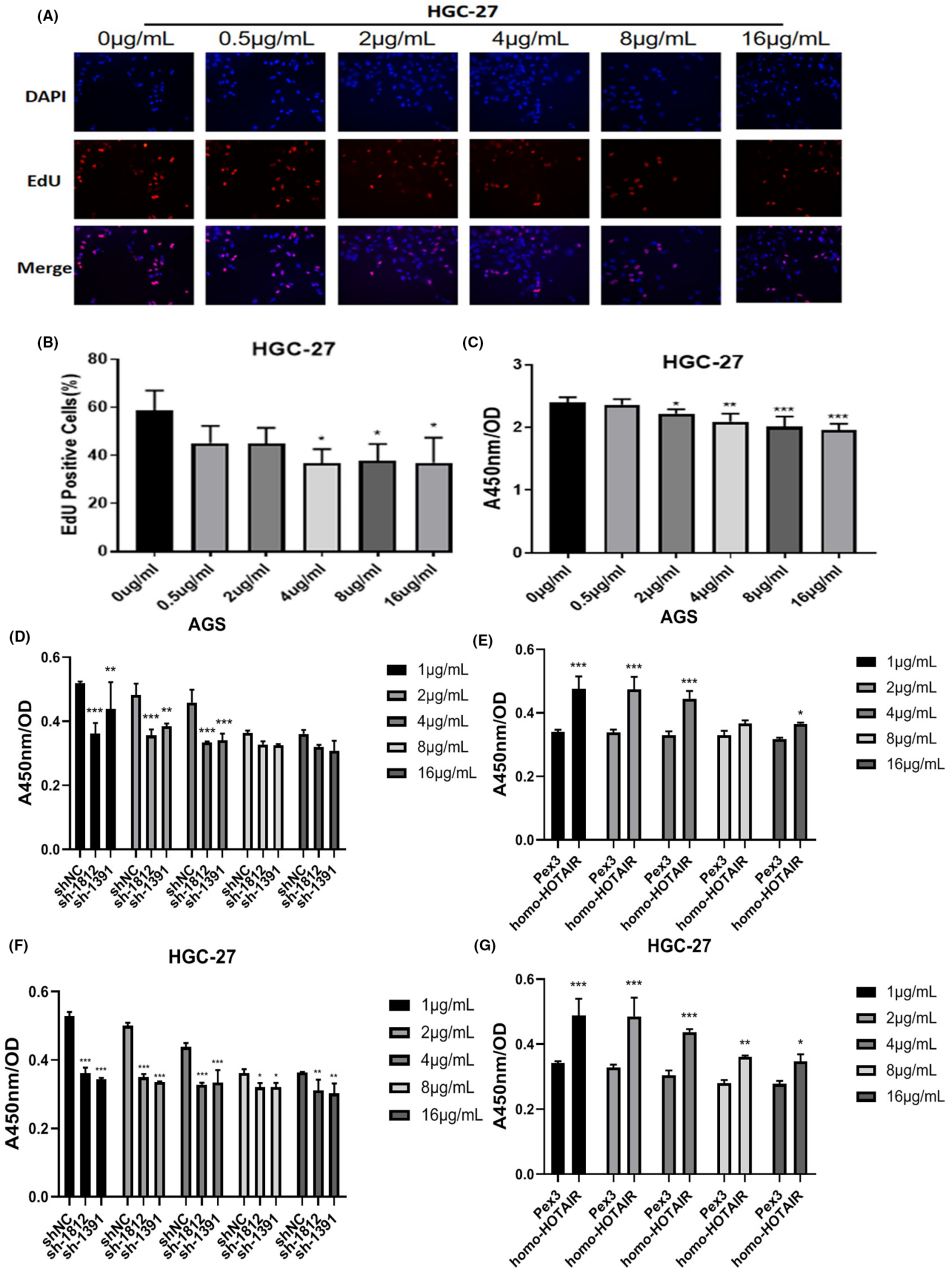
HOTAIR elevated the resistance of gastric cancer cells to oxaliplatin. (A–C) The survival of gastric cancer cells treated with increasing concentration of oxaliplatin. EdU incorporation (A, B), CCK‐8 assay (C). (D–G) CCK‐8 assay of AGS (D) and HGC‐27 (F) with HOTAIR knockdown and overexpression (E, G), treated with increasing concentration of oxaliplatin (*p < 0.05; **p < 0.01; ***p < 0.001).

**FIGURE 6:**
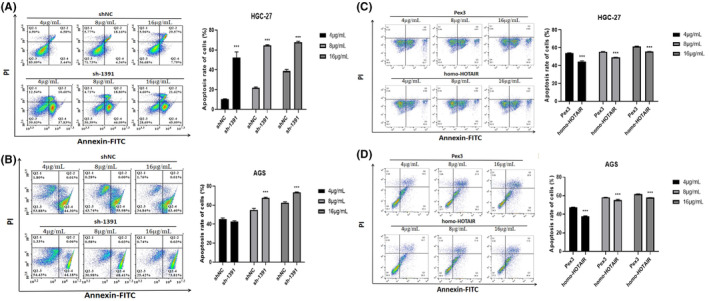
Apoptosis assay of HGC‐27 and AGS with HOTAIR knockdown (A, B) and overexpression (C, D), treated with increasing concentration of oxaliplatin. Data were expressed as mean ± SD (***p < 0.001).

We tested the role of HOTAIR in the regulation of GC cells resistance to oxaliplatin. When exposed to different concentration gradients of oxaliplatin, CCK‐8 assay indicated that the concentration of 4 μg/mL oxaliplatin suppressed the growth of GC cells (Figure 5A–C). Silencing HOTAIR significantly enhanced the sensitivity of GC cells to oxaliplatin (Figure 5D,F), while overexpression of HOTAIR presented the opposite effects in GC cells (Figure 5E, G). Moreover, silencing HOTAIR significantly enhanced the sensitivity of GC cells to oxaliplatin through apoptosis assay (Figure 6A–D, *p < 0.05; **p < 0.01;***p < 0.001). The above results showed that HOTAIR contributed to the proliferation and induced the insensitivity to oxaliplatin in GC cells.

### 3.4 HOTAIR acts as RNA sponge to downregulate miR‐195‐5p

**FIGURE 7:**
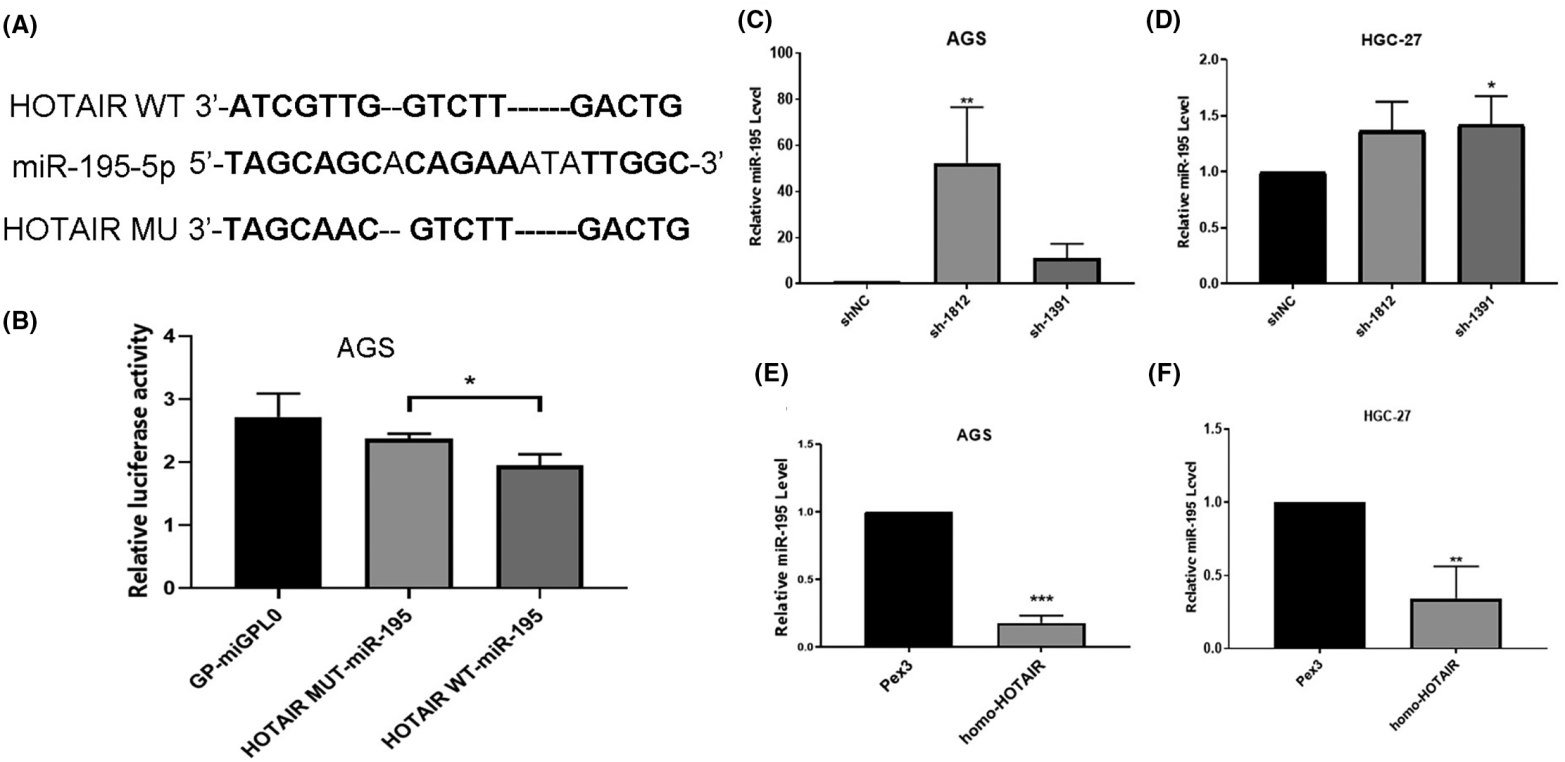
HOTAIR acts as a ceRNA and competitively sponge miR‐195‐5p. (A) The predicted miR‐195‐5p binding sites in HOTAIR. (B) The luciferase activities in gastric cancer cells. (C–F) The relative expression of miR‐195‐5p in AGS and HGC‐27 with HOTAIR knockdown (C, D) and overexpression (E, F).

**FIGURE 8:**
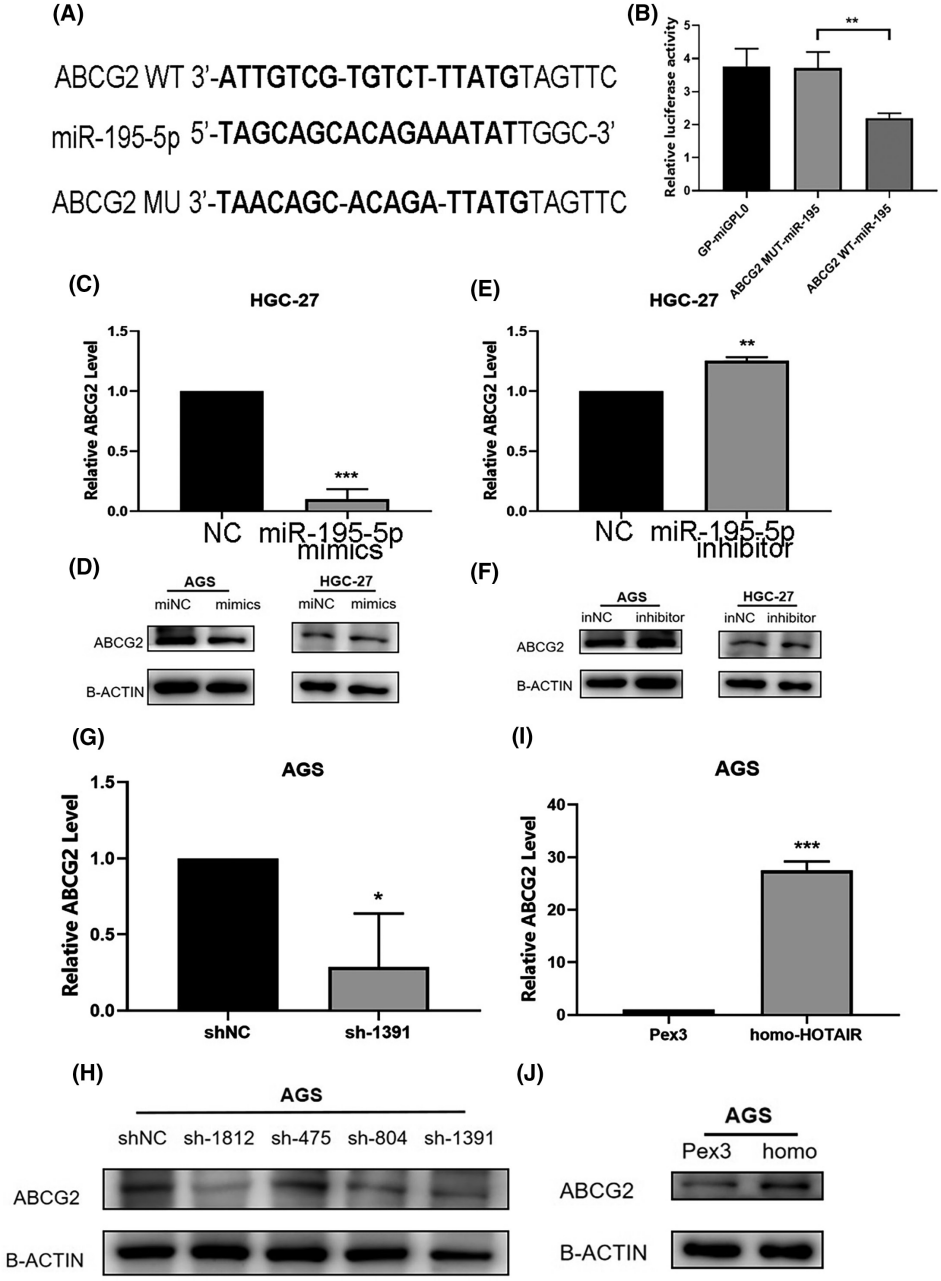
ABCG2 competitively sponges miR‐195‐5p. (A) The predicted miR‐195‐5p binding sites in ABCG2. (B) The luciferase activities in gastric cancer cells. (C–F) The relative expression of ABCG2 in gastric cancer cells transfected with miR‐195‐5p mimics (C, D) and inhibitors (E, F). (G–J) The relative expression of ABCG2 in gastric cancer cells with HOTAIR knockdown (G, H) and overexpression (I, J).

We used database including TargetScan, miRDB, miRBase, RNA Hybrid and DIANA to predict the targets of HOTAIR. We found that HOTAIR could bind to miR‐195‐5p. We also found that ABCG2 was the regulatory target of miR‐195‐5p (Figure 7A and Figure 8A). We constructed the dual luciferase reporter gene to evaluate the regulatory role of miR‐195‐5p (Figure 7B, Figure 8B). On the other hand, we further evaluated the opposite effects of miR‐195‐5p and HOTAIR by qRT‐PCR. The results showed that miR‐195‐5p was up‐regulated after down‐expression of HOTAIR while down‐regulated after over‐expression of HOTAIR (Figure 7C–F, *p < 0.05; **p < 0.01; ***p < 0.001). The results showed that the carcinogenetic role of HOTAIR was regulated by the suppression of miR‐195‐5p in GC.

### 3.5 HOTAIR relieves the inhibition of miR‐195‐5p on ABCG2

ABCG2 was reported to be closely linked with tumour progression and oxaliplatin resistance in many types of cancer. The expression of ABCG2 was suppressed by miR‐195‐5p but elevated by suppression of miR‐195‐5p (Figure 8C–F, **p < 0.01; ***p < 0.001). Furthermore, the expression of ABCG2 was upregulated after the overexpression of HOTAIR, similarly the ABCG2 was downregulated after silencing HOTAIR (Figure 8G–J, *p < 0.05; ***p < 0.001). The above results showed that HOTAIR upregulates ABCG2 and thus promote the resistance of GC cells to oxaliplatin via miR‐195‐5p.

### 3.6 ABCG2 is responsible for the HOTAIR‐regulated cell proliferation and oxaliplatin resistance

**FIGURE 9:**
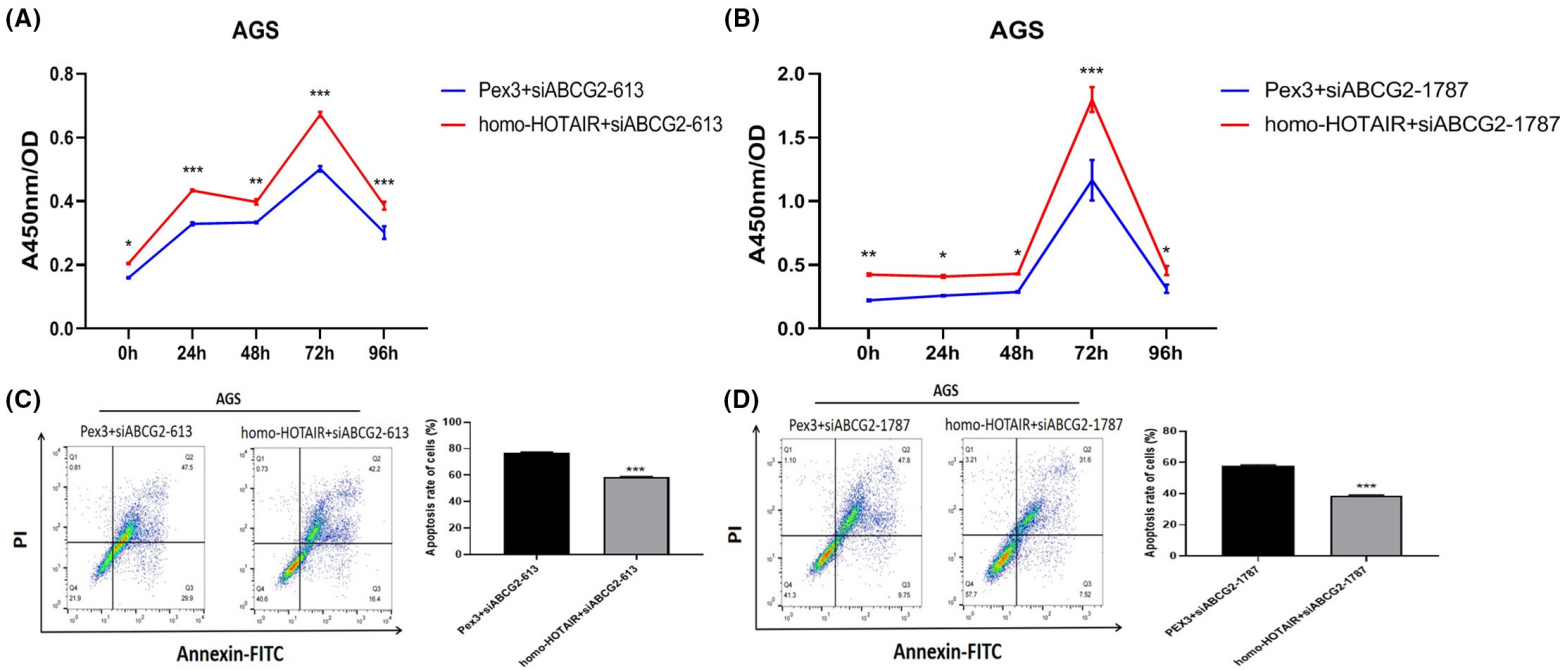
Silencing of ABCG2 partially reduced the proliferation and promoted apoptosis of AGS transfected with homo‐HOTAIR. (A, B) CCK‐8 assay showed that knockdown of ABCG2 partially reduced the proliferation of AGS transfected with homo‐HOTAIR. (C, D) Apoptosis assay showed that the elevated apoptosis rates of AGS in the same above groups.

To evaluate the HOTAIR‐regulated resistance of GC cells to oxaliplatin, CCK‐8 results showed that the silencing of ABCG2 partially reduced the proliferation of AGS transfected with homo‐HOTAIR and siABCG2 (Figure 9A,B). The apoptosis rates of AGS were increased after silencing of ABCG2 compared with those of cells transfected with homo‐HOTAIR and siABCG2 (Figure 9C,D). The above findings showed that ABCG2 was responsible for the HOTAIR‐regulated cell proliferation and oxaliplatin resistance.

## 4 DISCUSSION

Most patients with GC initially appeared to respond well to oxaliplatin. However, the resistance to oxaliplatin remains a major obstacle of therapy failure. It is of great importance to investigate the mechanism of oxaliplatin resistance. In the present study, we demonstrated that HOTAIR was overexpressed in GC and indicated a poor prognosis in patients with GC. We demonstrated that HOTAIR sponged miR‐195‐5p to facilitate ABCG2 expression and thus promote the proliferation of GC cells and the resistance to oxaliplatin. Thus, HOTAIR function as a prognostic biomarkers and therapeutic targets in GC.

Previous studies demonstrated that HOTAIR enhanced the resistance of colorectal cancer to oxaliplatin.^15^, ^16^, ^17^ To our knowledge, for the first time our evidence demonstrated that HOTAIR contributed to the resistance of GC to oxaliplatin dependent on ABCG2. These evidences indicated that HOTAIR could be a promising target for personalization chemotherapy for GC in future. A number of studies have demonstrated that the competing endogenous RNA network involved in many aspects of carcinogenesis.^18^, ^19^, ^20^, ^21^ With the bioinformatics predication, we confirmed that HOTAIR and ABCG2 can bind to miR‐195‐5p verified by functional experiments assay. The role of miR‐195‐5p in carcinogenesis remains unclear. To date, most of studies indicated that miR‐195‐5p served as a tumour suppressor gene in cancer progression.^22^, ^23^, ^24^, ^25^ For example, in GC, miR‐195‐5p was low expressed and regulated multi drug resistance by targeting ZNF139.^26^ Another study showed that circular RNA‐PVT1 modulates EST1 expression by competitively binding to miR‐195‐5p in GC.^27^ Patients with colorectal cancer had a low level of miR‐195‐5p presented shortened overall survival.^28^ Consistent with these studies, we found that the inhibiting effect of miR‐195‐5p in GC via ABCG2 pathway.

In the present study, we demonstrated that ABCG2 is the downstream target of HOTAIR. ABCG2 is the second member of subfamily G of the ABC transporter superfamily. Previous studies have demonstrated that several chemical agents such as DOX, irinotecan/CPT‐11 and epirubicin were all ABCG2's substrates.^29^ ABCG2 was an important gene involved in chemotherapy resistance of most cancer cells.^4^, ^30^, ^31^ To confirm if HOTAIR could regulate the expression of ABCG2, we knocked down HOTAIR and found that the expression of ABCG2 were significantly lowered (Figure 8G,H). The opposite effects were observed when overexpression of HOTAIR in GC cells (Figure 8I,J). To verify even miR‐195‐5p could regulate the expression of HOTAIR and ABCG2, the luciferase reporter gene results showed that miR‐195‐5p could specifically bind to both HOTAIR and ABCG2 (Figure 7A,B and Figure 8A,B). While we acknowledge the HOTAIR/miR‐195‐5p/ABCG2 axis in regulating oxaliplatin resistance, we also recognised that there might be other putative downstream signalling pathways of HOTAIR. For example, the recent study demonstrated that miR‐1227‐5p/ZEB1 as the downstream signalling pathway of HOTAIR in regulating oxaliplatin resistance.^17^ The reasons why the research above found different downstream pathways of HOTAIR were as follows. The authors used different cancer tissues and cells and different experimental conditions. They used colorectal cancer tissues and cells to find the potential downstream signalling pathways of HOTAIR, which leads to the different results. To exclude other putative downstream targets of HOTAIR, we intend to employ additional protein and nucleic acid assays in our future research.

The findings from this study carry significant clinical implications. First, HOTAIR exhibited markedly elevated expression levels in GC patients and displayed a strong correlation with unfavourable clinical prognosis. These observations indicate the potential utility of HOTAIR as a valuable biomarker for clinical diagnosis and prognostic evaluation in GC. Second, our research elucidated the mechanistic link between HOTAIR and oxaliplatin resistance in GC by functioning as a molecular sponge for miR‐195‐5p. This mechanistic insight sheds light on the underlying pathways through which HOTAIR contributes to the development of chemoresistance. Consequently, HOTAIR emerges as a promising therapeutic target for alleviating chemoresistance in the clinical management of GC.

In the present study, we demonstrated that HOTAIR upregulated the expression of ABCG2, which promoted the proliferation of GC cells and resistance to oxaliplatin. If ABCG2 was the unique pathway mediated by HOTAIR to promote the proliferation and oxaliplatin resistance of GC needs to be further investigated, and the downstream regulatory molecules needs to be further explored. Considering the extensive functions of HOTAIR in cells, a deeper understanding of how HOTAIR‐mediated oxaliplatin resistance will give us a new horizon for the treatment of GC.

## 5 CONCLUSION

In conclusion, the present study elucidated the function of HOTAIR in promoting proliferation and oxaliplatin resistance of GC cells by miR‐195‐5p/ABCG2 regulatory axis. Our results may facilitate an in‐depth understanding of the mechanism involved in oxaliplatin resistance of cancer and provide new basis to develop more effective therapeutic targets for chemotherapy in patients with GC.

## AUTHOR CONTRIBUTIONS

**Yaomin Luo:** Methodology (equal); validation (equal). **Xintong Lu:** Methodology (equal); validation (equal). **Wenrong Ma:** Methodology (equal); validation (equal). **Yang Xiao:** Methodology (equal); validation (equal). **Chen Wei:** Methodology (equal); validation (equal). **Xiaoxia Yuan:** Methodology (equal); validation (equal). **Yueyue Wu:** Investigation (equal); validation (equal). **Yunlin Wang:** Methodology (equal); validation (equal). **Yiman Xiong:** Investigation (equal); validation (equal). **Xin Yu:** Methodology (supporting). **Xue Wu:** Methodology (equal); validation (equal). **Siqi He:** Methodology (supporting); validation (supporting). **Yayudie Liu:** Validation (supporting). **Jinjing Wang:** Investigation (supporting); validation (supporting). **Qing Wu:** Investigation (supporting); validation (supporting). **Hui Zhou:** Validation (equal). **Zhen Jiang:** Conceptualization (equal); data curation (equal); formal analysis (equal); funding acquisition (equal); investigation (equal); methodology (equal); project administration (equal); resources (equal); software (equal); supervision (equal); validation (equal); visualization (equal); writing – original draft (equal); writing – review and editing (equal).

## FUNDING INFORMATION

The Sichuan Provincial Department of Science and Technology (2020YJ0379); The Special Foundation of Cooperation between Nanchong Government and North Sichuan Medical College (20SXJCQN0004; 20SXQT0053; 18SXHZ0281); The Science and Technology Project of Jiaxing City (2020AD30058);

Jiaxing Key Discipline of Medicine – Clinical diagnostics (2023‐ZC‐002).

## CONFLICT OF INTEREST STATEMENT

The authors declare no conflict of interest.

## Data Availability

The datasets used and/or analysed during the current study are available from the corresponding author on reasonable request.
